# Exotic plants introduction changed soil nutrient cycle and symbiotic relationship with arbuscular mycorrhizal fungi in wetland ecological projects

**DOI:** 10.3389/fpls.2024.1410009

**Published:** 2024-07-10

**Authors:** Yuxin Jiang, Mengxuan Wang, Xue Yan, Miaodan Liu, Xiaohong Guo

**Affiliations:** School of Resources and Environmental Engineering, Ludong University, Yantai, China

**Keywords:** exotic plants introduction, coastal wetland, *spartina alterniflora*, arbuscular mycorrhizal fungi, plant invasion

## Abstract

In the process of applying exotic plants to wetland ecological restoration, insufficiently evaluated alien species may exhibit strong competitiveness and fecundity. Once introduced, they can displace native flora, disrupt the original ecological balance, diminish biodiversity, and even induce ecosystem dysfunction. Furthermore, exotic plants have the potential to alter soil microbial community structure, including the composition and activity of beneficial symbiotic microorganisms such as arbuscular mycorrhizal fungi (AMF), thereby impacting soil nutrient cycling and interplant nutrient competition. Here, we conducted three consecutive years of sampling experiments to investigate the succession of AMF communities associated with the invasive plant *Spartina alterniflora* along an initial introduction chronosequence, and to identify the key environmental factors influencing its response to *S. alterniflora* invasion. Our findings reveal that early-stage invasion by *S. alterniflora* alters the composition of soil AMF communities with *unclassified_c__Glomeromycetes* and *Glomus-viscosum-VTX00063* consistently dominating. Additionally, as the duration of introduction increases, the diversity of rhizosphere soil AMF significantly decreases, while its evenness remains relatively stable. It’s indicated that soil ω, AN, AK and N/P ratio were the main influencing factors of the integral AMF community. Notably, soil available phosphorus (AP) emerges as a positive influence on the important AMF taxa. The results confirm the mutual feedback effect between the invasion of the perennial herb *S. alterniflora* and AMF, in which specific AMF assist in nutrient absorption to promote *S. alterniflora* growth, potentially facilitating its rapid and successful invasion of new habitats. Given the likely differential effects of AMF communities on various plant species, our findings could contribute to anticipating future AMF-mediated effects during the introduction of alien plants.

## 1 Introduction

Coastal wetlands are highly ecologically vulnerable, and the superimposed effects of global climate change and human activities have led to severe degradation of natural wetlands (Osland et al., 2022), an urgent issue that requires global focus and response. Vegetation plays a crucial role in wetland water purification, preservation, and regulation (Bhatia and Goyal, 2014). In wetland ecological restoration, it is imperative to rationally screen and utilize exotic plants (Herron et al., 2007). If the invasive potential of an introduced species is not properly assessed before it is introduced, it may become an invasive species in its new habitat, causing potential disruptions to ecosystems and economies (Pyšek and Richardson, 2010).

Nevertheless, the current studies are lacking in comprehensive case analyses and sufficient data, resulting in the uncertainty of the effectiveness of vegetation restoration strategies in enhancing the wetland ecological roles. Plant invasion has emerged as a driving force for global transformation, reshaping the structure and function of native ecosystems worldwide (Herron et al., 2007; Bunn et al., 2015). Evidence suggests that the penetration of plants across various ecosystems profoundly influences the microbial communities living beneath the surface and their associated ecosystem mechanisms (Fahey et al., 2020). Given that soil microbial communities can rapidly respond to external environmental changes (e.g., plant invasion) and are critical to soil nutrient dynamics, they can act in dual roles as drivers and responders for plant invasion (Dawson and Schrama, 2016). Lately, the mutual feedback between plant communities and soil microorganisms in coastal wetlands has received more attention. For instance, a study investigated the impact of different plant species on fungal changes in the coastal wetland of the Minjiang River Estuary (Ye et al., 2023), demonstrating that non-native species can increase fungal diversity, and the relative abundance of saprophytic and pathogenic fungi. Yu et al. (2012) and Li et al. (2021) examined the response of soil microbial communities to wetland vegetation succession in the Yellow River Delta, and revealed an obvious interaction between aboveground and underground components, demonstrating that wetland plants enhance the diversity of soil microorganisms.

Arbuscular Mycorrhizal Fungi (AMF) is capable of establishing symbiotic relationships with more than 80% of terrestrial plants (Smith, 2009). This mechanism sustains the equilibrium of the ecosystem by enhancing plant development through improved nutrient accessibility, thereby changing the competitive dynamics within plant communities (Chen et al., 2020). Many introduced plant species rely on mutualistic interactions to get over the obstacles in their new habitats and become naturalized, and even turn into invasive ones (Richardson et al., 2000). Mycorrhizal associations themselves, as along with fungal diversity and mycorrhizal types, directly or indirectly influence plant dispersal and competition, shaping plant populations and communities and regulating plant coexistence and diversity at local scales (Tedersoo et al., 2020). The response of soil AMF communities to alien plant invasion and the feedback of intrinsic soil AMF communities to plant invasion success have garnered increasing attention (Lekberg et al., 2013; Sun et al., 2022a). Some studies showed that AMF colonization rates and the average growth patterns remained similar in both native and invasive plant species, but AMF was more possible to affect invasion trajectories when native and invasive plants belong to different functional groups (Bunn et al., 2015). It is controversial whether there is a specificity regarding the associations between AMF and plants. Some researchers think there is low specificity, while specific research has indicated that the presence of host plant predilections could be critical in maintaining the diversity and abundance of AMF (Torrecillas et al., 2012).

Recent advances in extensive sampling and sequencing methods indicate that seasonality and temporal effects contribute to forming the AMF community (Davison et al., 2011; Han et al., 2016). The impact of plant invasion on ecosystems varies over time, and the initial advantage of invasive species may diminish in the later stages of the invasion (Dostál et al., 2013). Specifically, the effects of invasive species on soil properties and microbial structure and function do not necessarily remain constant or accumulate during the invasion process (Strayer, 2012; Souza-Alonso et al., 2015). Besides that, seasonality can be defined as periodic and cyclical changes in conditions over an annual time scale (Williams et al., 2017). Its impact on ecological processes has been fully recognized, but its role in ecosystems has not been fully valued (White and Hastings, 2020). Notably, previous research has demonstrated significant seasonal variations in soil microbial communities and their functional categories of invasive plant (Zhang et al., 2023). Consequently, given the critical role of AMF, disentangling the dynamics of soil AMF communities along the chronological sequence of invasion and seasonal changes can help to understand the interactions between invasive plants and AMF.

*S. alterniflora*, a perennial grass native to the Atlantic coast of North America and the Gulf of Mexico, was initially introduced to China as an ecological engineering solution to enhance wetland soil quality and foster ecological rejuvenation (Li et al., 2022). However, it has been one of the most serious invaders in salt marshes globally (Ning et al., 2019). Specifically, in China, the largest colonization of *S. alterniflora* to date has been observed in coastal wetlands between 18°N and 41°N (Li et al., 2022). *S. alterniflora* invades the ecological niche of native species, leading to a sharp decline in native plant diversity and ecosystem functions (Wan et al., 2009; Dou et al., 2022; Li et al., 2022), and strongly altering the structure and composition of soil microbial communities (Fahey et al., 2020). While some studies have highlighted shift in microbial communities associated with *S. alterniflora* invasion, the function of mutualists (AMFs) in promoting or inhibiting its invasions (i.e., the capability of AMFs to affect the invasion process) remains uncertain (Richardson et al., 2000). Given the variability in how host plants affect distinct AMF (Šmilauer et al., 2020), it is crucial to comprehend how *S. alterniflora* modifies AMF communities in invaded soil.

In summary, it is of major value to elucidate the shifts in AMF communities during *S. alterniflora*’s invasion chronosequence and the coupling relationship between these changes and soil environmental factors. So, we assessed alterations of soil AMF communities following *S. alterniflora* invasion in a growing invasive saltmarsh habitat. The composition and structure of soil AMF communities were determined via 18S rRNA gene high-throughput sequencing technology. This study proposes and tests the following hypotheses: (1) *S. alterniflora* invasion will change the AMF diversity and community structure of coastal salt marshes over a short-term period of continuous invasion with seasonal differences; (2) the significant differences in AMF community structure and composition in the early stage of the invasion are closely tied to changes in soil characteristics.

## 2 Materials and methods

### 2.1 Study sites and soil sample collection

The invaded territory in the salt marshes (37°16′N, 118°59′E) of *S. alterniflora*, situated in the coastal region of Laizhou Bay, the Yellow River Delta of China, was chosen as the study location. It features a temperate continental monsoon climate, whose average annual temperature is 11.9°C, with approximately 592 mm of precipitation and more than 1900 mm of evaporation yearly. The main vegetation types include *S. alterniflora*, *Tamarix Chinensis*, *Suaeda salsa* and so on. Based on field surveys, reviews of the literature and remote sensing photos of the region, it was concluded that *S. alterniflora* invaded the sample site around 2017. A 5 m×5 m sample plot was set up in the invasion area for soil sampling, and samples were obtained using a five-point sampling method. Before sampling, the soil was manually removed of plant residues and visible stones. Based on the above determined locations, surface layer (0~20 cm) soil samples were gathered in April and November 2017, April and June 2018, and April and November 2019, for a total of 18 samples. Each sample was put in a sterile polyvinyl chloride bag and kept cold in an ice-filled refrigerator. For microbial community analysis, one component of each composite sample was brought to the lab and kept in a fridge maintained at -80°C. The other component was allowed to air dry at room temperature and sieved for soil physicochemical analysis.

### 2.2 Soil physicochemical analysis

Soil physical and chemical features were analyzed using established procedures. Soil moisture (ω) was calculated based on the water content loss after drying 10 g wet soil at the oven with 105°C (Gardner, 1986). Briefly, the supernatant of the soil sample (derived from a soil-water blend with a 1:5 weight ratio) was analyzed to determine its pH level and electrical conductivity (Ec, indicating soil salinity). Soil available phosphorus (AP) was measured by treatment with 0.5 mol/L NaHCO_3_ followed by molybdenum blue colorimetry. Soil available nitrogen (AN) was measured by the alkaline hydrolysis diffusion method. Soil available kalium (AK) was determined by ammonium acetate extraction with the flame photometry method. The wet combustion analysis of soils by chromic acid digestion was used to determine soil organic carbon (SOC) (Nelson and Sommers, 1996). Soil total nitrogen (TN) was determined by the micro-Kjeldahl method (Bremner, 1965). Soil C/N ratio and N/P ratio was calculated based on the SOC and TN molar content, and the AN and AP molar content, respectively.

### 2.3 DNA extraction, amplification and MiSeq sequencing

Following the manufacturer’s protocol, soil microbial genomic DNA was extracted from 0.5 g fresh soil using the E.Z.N.A.^®^ Soil DNA Kit (Omega Bio-Tek),. The measurement of DNA length and impurities was conducted on a 1% agarose gel, while the NanoDrop 2000 UV-vis spectrophotometer was employed to evaluate the DNA’s concentration and purity (Thermo Scientific, Wilmington, Delaware, USA). To assess AM fungal communities, the 18S rRNA gene fragment were nested polymerase chain reaction (PCR) amplified with the primer pair AML1 (5′-ATC AAC TTT CGATGG TAG GAT AGA-3′) and AML2 (5′-GAA CCC AAA CAC TTT GGT TTC C-3′) (Lee et al., 2008), and the second round primers were AMV4.5NF (5′-AAG CTC GTA GTT GAA TTT CG-3′) and AMDGRR (5′-CCC AAC TAT CCC TAT TAA TCA T-3′) (Sato et al., 2005). PCR results were further purified and paired-end sequencing was carried out using an Illumina MiSeq PE300 platform (Illumina, San Diego, CA, USA) in Majorbio Bio-Pharm Technology (Shanghai, China).

### 2.4 Bioinformatic analyses

Initial AMF sequencing outcomes underwent demultiplexing, quality standardization through fastp version 0.20.0 (Chen et al., 2018), and were combined using FLASH version 1.2.7 (Magoč and Salzberg, 2011). Using UPARSE version 7.1.1090 (Edgar, 2013), sequences that met the criteria were categorized into operational taxonomic units (OTUs) based on 97% similarity level, succeeded by pinpointing and omitting chimeric sequences. Each OTU representative sequence’s classification was scrutinized using the MaarjAM database (https://maarjam.botany.ut.ee/). To standardize the survey effort, we conducted a random resampling of 2434 reads in each sample and the diversity indices were determined based on this standardized data set.

### 2.5 Statistical analyses

Soil microbial alpha diversity was determined through the “vegan” software in the R (version 4.0.2). The assessment of microbial beta diversity utilized the weighted UniFrac distance, alongside a non-metric multidimensional scaling (NMDS) ordination to explore the fluctuations in microbial populations over various years or seasons. The impact of yearly and seasonal variations on the physicochemical characteristics of soil was analyzed using two-way ANOVA. To assess the variance in alpha diversity across different years or seasons, a one-way ANOVA with Tukey’s test was utilized. PERMANOVA was employed to evaluate how plant invasion timelines and seasonal patterns affect microbial communities. Kruskal-Wallis H test was used to analyze the significant difference among AMF communities in interannual and seasonality. Redundancy analysis (RDA) was employed to examine connection between microbial populations and environmental elements. Mantel test in “vegan” package’s mantel function was employed to investigate possible soil-related factors influencing microbial community changes, with Spearman correlation to analyze the relationship between AMF communities and environmental factors. Partial least squares structural equation modeling (PLS-SEM) was utilized to study possible causal relationships between physicochemical properties and related AMF community composition in IBM SPSS Amos Graphics (version 25).

## 3 Results

### 3.1 Soil physicochemical properties

*S. alterniflora* invasion affects soil physical and chemical properties along interannual time series and seasonal changes (**Table 1**). Two-way ANOVA showed that soil pH, AN, TN, AP, AK and SOC in wetland salt marshes changed significantly among interannual chronosequence or seasonality. Soil AN and AP were significantly reduced during the year of invasion, while soil pH and N/P ratio significantly increased. Lower values of soil AN, TN, AP, AK and SOC and higher C/N ratio and N/P ratio were observed in June, all of which changed significantly. Invasion chronosequence and seasonality had a significant interaction effect on soil pH, TN, AP, AK, SOC, C/N ratio and N/P ratio.

**Table 1:** Soil physicochemical properties of different sampling sites in the invaded salt marshes.

Year	Season	Soil moisture (ω) (%)	Soil pH	Soil Ec (μs cm^-1^)	AN (mg kg^-1^)	TN (g kg^-1^)	AP (mg kg^-1^)	AK (mg kg^-1^)	SOC (g kg^-1^)	C/N ratio	N/P ratio
2017	April	22.10 ± 0.51	7.41 ± 0.07	2714.44 ± 249.40	22.92 ± 1.04	0.72 ± 0.03	25.35 ± 1.60	175.19 ± 13.05	1.25 ± 0.07	1.72 ± 0.03	0.91 ± 0.06
	November	24.01 ± 0.45	7.70 ± 0.02	2714.44 ± 249.41	25.73 ± 2.44	0.63 ± 0.57	18.91 ± 0.80	281.65 ± 2.63	2.38 ± 0.19	3.86 ± 0.51	1.36 ± 0.12
2018	April	23.13 ± 0.34	7.71 ± 0.13	3483.45 ± 161.86	25.72 ± 1.64	0.40 ± 0.01	24.08 ± 0.24	273.01 ± 14.48	2.36 ± 0.20	5.95 ± 0.49	1.07 ± 0.06
	June	22.12 ± 0.90	7.51 ± 0.03	4594.44 ± 571.37	12.17 ± 0.67	0.13 ± 0.00	8.71 ± 0.12	140.52 ± 18.78	1.30 ± 0.07	10.40 ± 0.53	1.39 ± 0.06
2019	April	21.18 ± 0.37	7.83 ± 0.07	3083.34 ± 236.67	16.76 ± 1.55	0.27 ± 0.08	9.31 ± 1.30	249.88 ± 11.73	1.52 ± 0.15	6.47 ± 1.15	1.84 ± 0.20
	November	22.43 ± 1.25	7.64 ± 0.04	3128.67 ± 189.88	18.32 ± 0.95	0.42 ± 0.09	13.93 ± 1.19	165.70 ± 1.83	1.34 ± 0.12	3.59 ± 0.86	1.35 ± 0.06
Two-way ANOVA	Year	ns	*	ns	***	***	***	*	***	**	**
	Season	ns	ns	ns	*	*	***	***	***	**	ns
	Year*Season	ns	**	ns	ns	*	***	***	***	**	**

The effect of invasion chronosequence and seasonality on soil parameters was tested by two-way ANOVA (***p < 0.001; **p < 0.01; *p < 0.05; ns *p*> 0.05). ω, soil water content; pH; Ec, soil electrical conductivity; AN, alkaline nitrogen; TN, soil total nitrogen; AP, soil available phosphorus; AK, soil available potassium; SOC, soil dissolved organic carbon; C/N, carbon to nitrogen ratio; N/P, nitrogen to phosphorus ratio.

### 3.2 Soil AMF diversity and community

In total, soil AMF sequences were clustered into 1 phylum, 1 class, 4 orders, 4 families, 4 genus, 15 species, and 158 OTUs. Venn plot was used to calculate the number of shared and unique species in a sample and analyze the AMF community composition in the rhizosphere of *S. alterniflora*. It showed a downward trend in the interannual series, but there still existed 50 OTU overlaps between three years, accounting for 31.65% (**Figure S1**).

**Figure 1:**
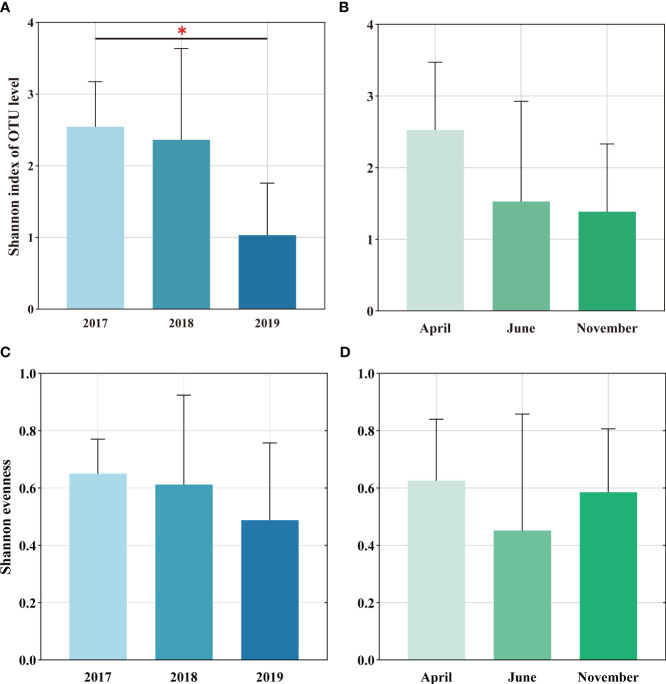
Soil AMF alpha diversity at invaded sites, including diversity **(A, C)** and evenness **(B, D)** of inter-annual sequence and seasonality, respectively. The differential representation between them was assessed by one-way ANOVA: * p <0.05.

Shannon diversity index of soil AMF in 2019 was significantly lower than that in 2017 (**Figure 1A**), while Shannon index showed a decreasing trend in seasonal changes (**Figure 1B**). Moreover, it was observed that Shannon evenness index did not change much over time, indicating that the evenness of the AMF community was relatively stable (**Figures 1C, D**).

**Figure 2:**
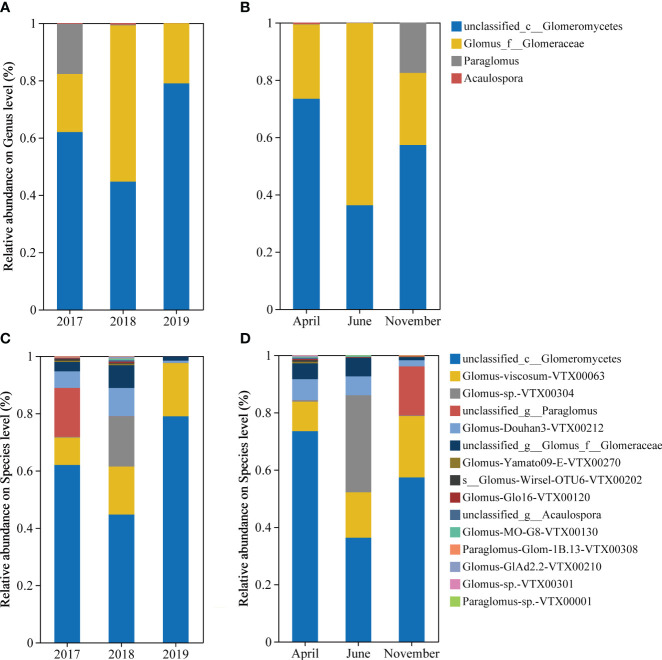
Characterization of AMF community composition. It includes the community composition of inter-annual sequence **(A)** and seasonal **(B)** at the genus level, and the community composition of inter-annual sequence **(C)** and seasonality **(D)** at the species level. AM fungal OUT with a relative abundance below 0.1% was classified as “other”. The category “Unclassified Fungi” indicates that these OTUs cannot be assigned a taxonomic affiliation based on the taxonomic database.

Soil AMF community composition also showed strong changes. On genus level (**Figures 2A, B**), *unclassified_c__Glomeromycetes* and *Glomus-viscosum-VTX00063* accounted for the majority, with *Paraglomus* accounting for 17.92% only in November 2017. At the species level, in terms of inter-annual community composition (**Figure 2C**), *unclassified_c__Glomeromycetes* and *Glomus-viscosum-VTX00063* are the dominant taxa. The proportions of *unclassified_g__Paraglomus* and *Glomus-sp.-VTX00304* were higher in 2017 and 2018, respectively. *unclassified_c__Glomeromycetes* and *Glomus-viscosum-VTX00063* are the dominant taxa in April and November, while the proportion of *Glomus-sp.-VTX00304* can reach more than 30% in June, and the proportion of *unclassified_g__Paraglomus* increases significantly in November (**Figure 2D**).

**Figure 3:**
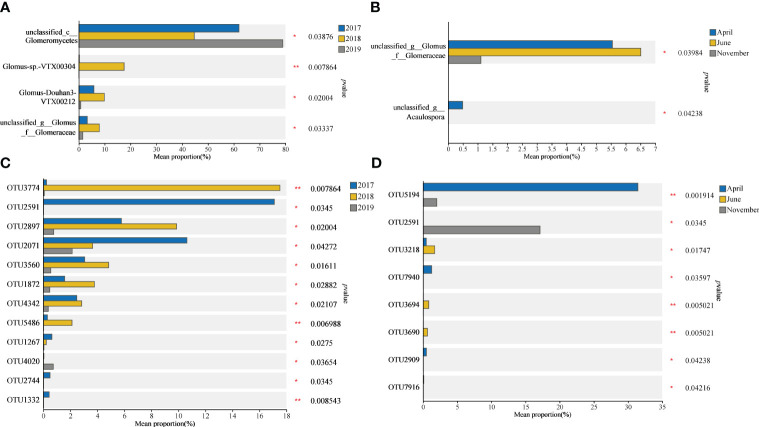
Difference significance test analysis between the most abundant species **(A, B)** and OUTs **(C, D)** in AMF community. The value of mean relative abundance indicates the species and OTU content of the AMF community in the rhizosphere of *S. alterniflora*. Statistical analyses were performed using the Kruskal-Wallis H test. (*p < 0.05, ** *p <*0.01).

Difference significance test analysis was used to compare groups of dominant species (**Figures 3A, B**) and dominant OTUs (**Figures 3C, D**) in order to determine the inter-annual (2017, 2018, and 2019) and seasonal (April, June, and November) differences in rhizosphere AMF of *S. alterniflora*. Findings indicated a notable reduction and subsequent rise in the quantity of *unclassified_c__Glomeromycetes* in *S. alterniflora* annually during the initial phases of invasion. The abundance of *Glomus-Douhan3-VTX00212* and *unclassified_g__Glomus_f__Glomeraceae* both increased significantly by nearly 2 times in 2018 compared to 2017 and fell to 8% -20% in 2019 compared to 2018, and the abundance of *Glomus-sp.-VTX00304* in 2018 was significantly higher than other years (**Figure 3A**). Seasonally, the abundance of *unclassified_g__Glomus_f__Glomeraceae* significantly decreased by approximately 80% in November, and *unclassified_g__Acaulospora* appeared in April (**Figure 3B**). Additionally, there are notable variations in the *S. alterniflora* soil AMF between years, in which the abundance of OTU2591, OTU2744, and OTU1332 dropped sharply in 2018, while the abundance of OTU2071 decreased directly year by year (**Figure 3C**). From the perspective of seasonal changes, OTU7940, OTU2909 and OTU7916 appeared in April (**Figure 3D**).

NMDS plots based on Bray-Curtis and weighted UniFrac distances showed differences in AMF community structure in invaded site soils across years and seasons (**Figure S2**). Soil AMF communities were significantly separated in different years and seasons, and this separation was confirmed by permutation tests (**Table 2**).

**Table 2:** The results of the Adonis/ANOSIM test with 999 permutations to indicate the effect of seasonality and interannual on soil AMF communities.

Method		Effect of interannual					Effect of seasonality			
		2017	2018	2019	Total		April	June	November	Total
Adonis Statistic (*p* value)	2017	/	/	/	0.224 **(0.001)**	April	/	/	/	0.213 **(0.001)**
	2018	0.140 **(0.020)**	/	/		June	0.228 **(0.021)**	/	/	
	2019	0.168 **(0.021)**	0.191 **(0.012)**	/		November	0.168 **(0.008)**	0.037 (0.369)	/	
ANOSIM Statistic (*p* value)	2017	/	/	/	0.460 **(0.001)**	April	/	/	/	0.186 **(0.001)**
	2018	0.170 **(0.034)**	**/**	/		June	0.485 **(0.030)**	/	/	
	2019	0.136 (0.096)	0.200 **(0.013)**	/		November	0.368 **(0.005)**	0.140 (0.298)	/	

The bold value indicates that p is less than 0.05 and is significant.

### 3.3 Microbial key drivers under the invasion

**Figure 4:**
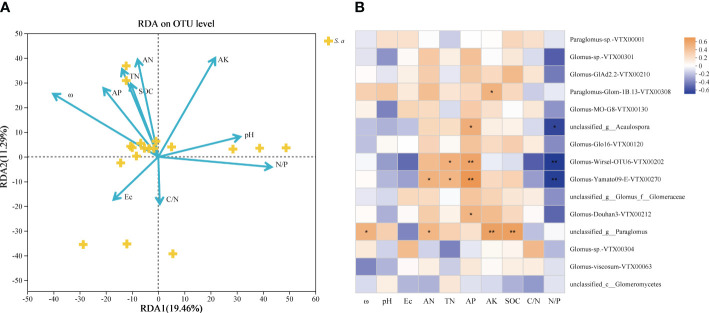
**(A)** Redundancy analysis (RDA) of soil AMF communities and soil physicochemical properties invaded by *S. alterniflora*. **(B)** Correlation heatmap of the influence of different environmental factors on the Species level community composition of soil AMF. ω, soil water content; pH; Ec, soil electrical conductivity; AN, alkaline nitrogen; TN, soil total nitrogen; AP, soil available phosphorus; AK, soil available potassium; SOC, soil dissolved organic carbon; C/N, carbon to nitrogen ratio; N/P, nitrogen to phosphorus ratio. * p <0.05; ** p < 0.01.

Redundancy analysis (RDA) outcomes reveal that the study attributes 30.75% of the total fluctuation in soil AMF communities to 10 pertinent environmental elements (**Figure 4A**). The results showed that soil ω, AN, AK and N/P ratio were the main influencing factors of the integral AMF community (**Table 3**). Additionally, the heatmap revealed that soil environmental variables impacted the 15 most prevalent AMF species (**Figure 4B**), where soil AP all showed significantly positive correlations with *unclassified_g__Acaulospora*, *Glomus-Wirsel-OTU6-VTX00202*, *Glomus-Yamato09-E-VTX00270* and *Glomus-Douhan3-VTX00212*.

**Table 3:** Environmental explanation of the changes in soil AMF community by RDA analysis.

	RDA1	RDA2	R^2^	*p*
ω	-0.820	0.572	0.505	**0.005**
pH	0.987	0. 160	0.198	0.190
Ec	-0.698	-0.716	0.105	0.426
AN	-0.285	0.959	0.340	**0.041**
TN	-0.428	0.904	0.314	0.063
AP	-0.620	0.785	0.269	0.097
AK	0.418	0.908	0.385	**0.028**
SOC	-0.403	0.915	0.211	0.177
C/N	0.132	-0.991	0.071	0.580
N/P	0.981	-0.195	0.390	**0.024**

The bold value indicates that p is less than 0.05 and is significant.

**Figure 5:**
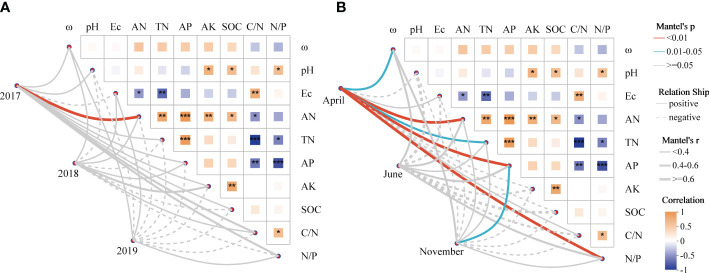
The relationship between soil AMF community turnover and soil parameters revealed by Mantel test. **(A)** The relationship between AMF beta diversity and soil variables in interannual sequences; **(B)** The relationship between AMF beta diversity and soil variables in seasons. Microbial and soil dissimilarity was assessed by Bray-Curtis distance. Edge width is proportional to Mantel’s *p*-value, and edge color indicates statistical significance. Pairwise correlations of soil variables were evaluated by Spearman correlations and visualized by heat maps with color gradients (correlation coefficients from -1 to 1 and colors from yellow to dark blue, respectively). * p < 0.05; ** p < 0.01; *** p < 0.001.

On account of the correlation heatmap of soil physicochemical factors, the Mantel test showed that soil AN was the main factor controlling the soil AMF community in 2017 in terms of interannual changes, and there was less connection between the AMF community and soil parameters in the following 2 years (*p* > 0.05; **Figure 5A**). Based on seasonal variations, soil ω, AN, TN, AP and N/P ratio were the main factors controlling the soil AMF community and had significantly positive effects in April; while only soil AP controlled the community significantly in November (**Figure 5B**).

**Figure 6:**
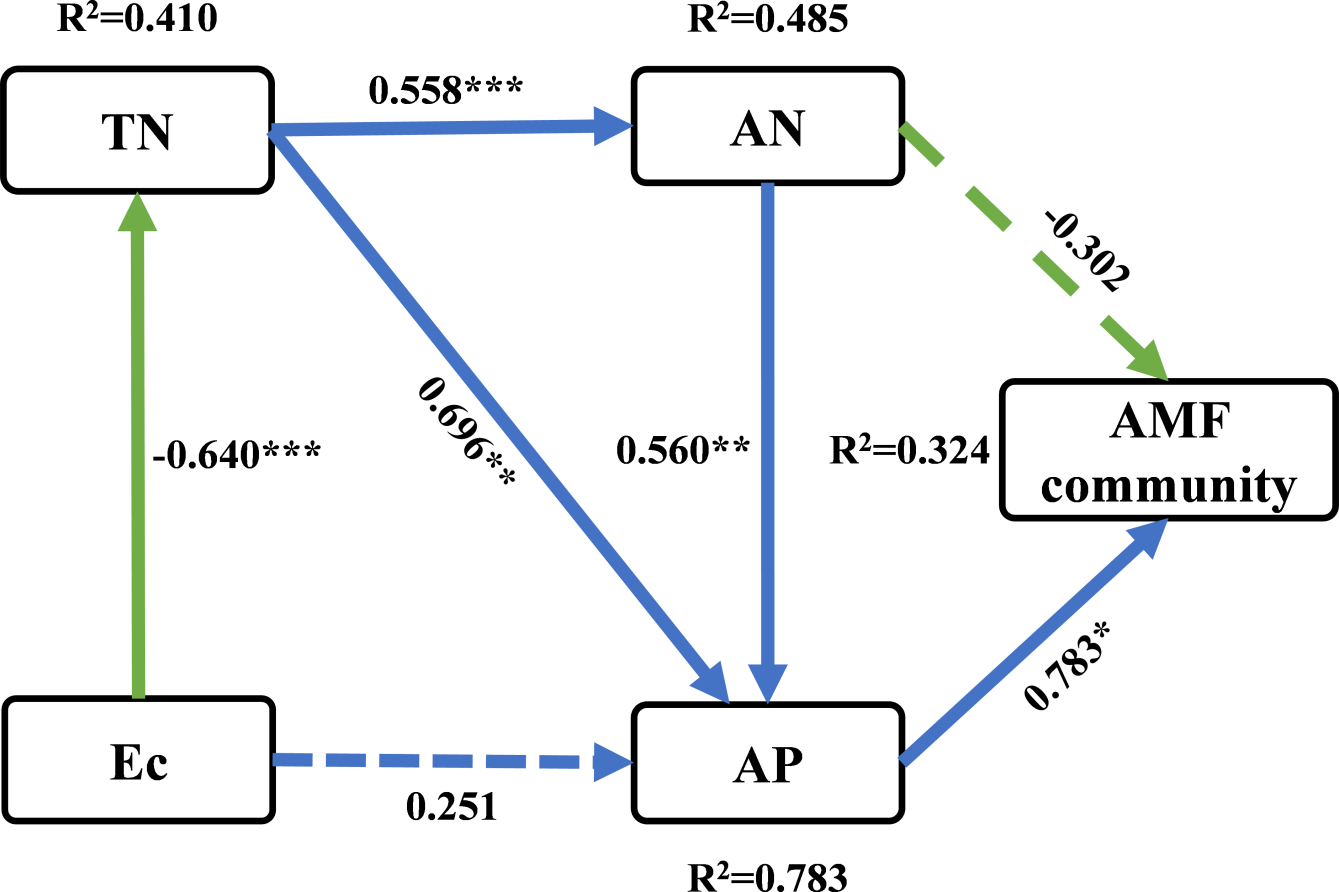
Partial least squares path modeling (PLS-SEM) was used to investigate the direct and indirect effects of soil physicochemical properties on soil AMF communities. The numbers on the arrows are standardized path coefficients. Solid arrows indicate significant effect sizes (*p* < 0.05, dashed lines *p* > 0.05), and blue and green colors indicate positive and negative relationships, respectively. The path coefficients and coefficients of determination (R^2^) were calculated after 999 bootstraps. * *p* < 0.05, ** *p* < 0.01, *** *p* < 0.001. R^2^, variance explained by the model.

In order to elucidate the changing patterns of soil AMF communities, a PLS-SEM model was established to quantitatively compare the causal relationship between multiple environmental variables and AMF communities. The PLS-SEM determined the direct and indirect effects of soil chemical properties (Ec, TN, AN and AP) on AMF communities (including all species) (**Figure 6**). TN demonstrated a notable positive association with AN and AP, respectively. AP was significantly positively correlated with soil AMF communities.

## 4 Discussion

### 4.1 Dynamic changes in soil physical and chemical properties

Plant invasion can change the biotic and abiotic properties of soil through positive or negative plant-soil feedback effects, thereby affecting the soil physical and chemical properties of the invaded habitat (Liao et al., 2006; Sun et al., 2022b). Under *S. alterniflora* invasion, the results of this study showed that some soil variables changed in an orderly manner during the invasion time series (**Table 1**). For example, with the growth of *S. alterniflora*, soil AN and AP significantly decreased, while pH and N/P ratio increased significantly. In the context of global change, the success of invasive plant species will also have an impact on the ability of wetlands to store and release nitrogen and phosphorus (Wang et al., 2014, Wang et al, 2018), and changes in nitrogen to phosphorus ratios have been shown to be associated with changes in plant performance and vegetation species composition (Güsewell, 2004). It is worth noting that *S. alterniflora* invasion is considered a key event affecting sediment P availability and storage, and changes in P content corresponding to *S. alterniflora* invasion depend on the invasion time (Li et al., 2018; Xie et al., 2019). For example, in the first stage of invasion, *S. alterniflora* mainly consumes organic phosphorus in sediments (Li et al., 2018). The similar phenomenon about AP was also observed in the study of Lin et al. (2024), in which a significant decreasing trend in AP was observed in the Minjiang estuary wetland invaded by *S. alterniflora*. The results of this investigation showed that more soil AP was lost in *S. alterniflora* soil than AN, with the soil N/P ratio rising and both AN and AP declining. According to the study of Wang et al. (2018), after *S. alterniflora* replaced *Phragmites australis*, soil phosphorus storage generally increased, but nitrogen storage did not increase, resulting in a decrease in the N/P ratio of soil and plants. The reasons for the inconsistency with our study may be the differences in native plants, the possibility of removal and replanting of *S. alterniflora* (Wang et al., 2015), or the invasion history of this site for 7–15 years. In addition, AMF is also one of the important driving factors affecting phosphorus components under *S. alterniflora* invasion (Zhang et al., 2024).

*S. alterniflora* is a polyploid grass (Ainouche et al., 2009), and its impact is likely to increase over time (Strayer, 2012). In addition, we surmise that the AN, TN, AP, AK and SOC contents of salt marsh soil showed a downward trend from April to June, and then increased again in November, with significant changes (**Table 1**). *S. alterniflora* produces overwintering ramets in autumn and winter, so this phenological difference provides *S. alterniflora* with a competitive advantage (Li et al., 2009), and in spring and summer, higher stem densities are observed due to the germination of new seedlings (Wan et al., 2009). It can be predicted that *S. alterniflora* is nutrient-dependent in the early stage of invasion and absorbs more nutrients during the greening period in spring (April) to the vigorous growth period (June) than in other periods of the year. In autumn, stems become stable and vegetation reaches top height (Wan et al., 2009), thus soil nutrients accumulate. In conclusion, the invasion of *S. alterniflora* leads to significant alterations in soil nutrient dynamics and plant-soil interactions, with implications for wetland ecosystem functioning and the success of the invasive species, especially considering its nutrient-dependent growth patterns and phenological advantages.

### 4.2 AMF community succession over the invasion time

The direction and extent of how plant invasion affects soil microbial variety and the makeup of communities depends on various elements such as the type of invasive plants, invasion intensity, invasion history and soil nutrient substrate (Strayer, 2012; Fahey et al., 2020; Bickford et al., 2022). The effectiveness of plant species invasions may be linked to their mutualistic associations with mycorrhizal fungi. In general, the thicker the root, the weaker its own absorption capacity, and it mainly relies on symbiotic mycorrhizal fungi to obtain nutrients. In this study, the Shannon index decreased slightly in the time series, but the Shannon evenness index did not change much (**Figure 1**), and the AMF community composition OTU gradually decreased (**Figure S1**). Previous studies have shown that significant AMF community changes and diversity reduction will occur after *Centaurea maculosa* invasion (Mummey and Rillig, 2006). Fungal richness, particularly symbiotic ones, decreased after two grass species (Bromus and Oats) invaded native shrubland in southern California, possibly by increasing their enhanced nutrient absorption in the soil compared to indigenous plants (Phillips et al., 2019). The above studies are consistent with ours (**Figure 1**). Such changes in AMF diversity could have significant impacts on above- and below-ground biodiversity. One study observed that invasion by *Reynoutria Japonica* reduced AMF spore numbers, species richness, and biomass over time (Zubek et al., 2022). Additionally, Lekberg et al. (2013) pointed out that compared with invasion species *Centaurea Stoebe* and *Euphorbia esula*, the enrichment of AMF in invasive *Bromus Tectorum* is low. This may be due to the reduced AMF richness in grass-dominated communities. AMF diversity varies in response to invasion, and some of the differences in these studies may be due to the mycorrhizal status of invaders. Additionally, 78% of meta-analyses (including wetland, grassland, and forest ecosystems, etc.) showed that invasive species harbored unique AMF communities (Bunn et al., 2015). Our study also found that the soil AMF community structure under *S. alterniflora* invasion exhibits significant interannual and seasonal changes (**Figure S2**, **Table 2**), which suggests changes in AMF composition and abundance are important for the ecosystem processes it mediates. Day et al. (2015) showed that for each site under *Vincetoxicum rossicum* invasion, the invasion explained a significant proportion of the variation in AMF composition over time, potentially leading to specific AMF communities. This study is consistent with our findings, but it does not rule out the possibility that different plant species have varied needs and responses to mutualistic communities, as well as the impact of plot history or soil texture on AMF communities. Consequently, S*. alterniflora* invasion influences soil AMF communities, and more research is needed to reveal whether AMF community changes during the early stages of growth contribute to *S. alterniflora*’s ability to compete relative to native plants when colonizing new areas.

### 4.3 Relationship between soil parameters and AMF communities

The dynamic turnover of microorganisms caused by plant invasion is intricately linked with the time series changes in soil characteristics, leading to significant shifts in microbial-driven ecological activities. *S. alterniflora* invasion can affect the soil microbial community structure by altering the available nutrients and soil physicochemical properties (Yang et al., 2016; Zhao et al., 2019). Our findings suggest that the primary determinants of alterations in AMF communities could be the soil ω, AN, AK and the N/P ratio (**Figure 4A**, **Table 3**). Variations in nutrient levels, particularly phosphorus and nitrogen, critically affect the AMF communities (Püschel et al., 2017; Treseder et al., 2018), potentially leading to shifts in the competitive edge of mycorrhizal invasive plants (Lin et al., 2015). Furthermore, evidence suggests that the balance between N and P is a more important factor in controlling fungal colonization than the effects of N or P alone (Miller et al., 2002). Wu et al. (2023) discovered a significant association between the composition of the soil AMF community and metrics like soil ω, AP, TN, and AN: AP, etc. The PLS-SEM we conducted further showed that AP showed a significant positive effect (0.783) on the AMF community (**Figure 6**). Liu et al. (2021) proved that under the invasion of the alien plant *Galinsoga quadriradiata*, soil AP concentration has a positive effect on root AMF colonization. If grown in soils under competition, fungal biomass and functional responses often depend on soil P and N:P, and it has been confirmed that AP changes the impact of AMF on native and invasive plants, which in turn affects their competition (Chen et al., 2020). These results support the findings that nutrient sources, temperature and moisture status interactions indirectly drive the distribution of soil AMF abundance and diversity nutrient source (Sun et al., 2018). Consequently, a comprehensive understanding of the ways in which AMF communities respond to and interact with their abiotic environment, specifically the availability of key soil nutrients, is indispensable for accurately predicting the trajectory of microbial community transitions and their wide-ranging ecological implications. This finding enables to inform targeted interventions to mitigate the impacts of invasive species, and ultimately foster more resilient native ecosystems in the face of ongoing biotic invasions.

## 5 Conclusion

**Figure 7:**
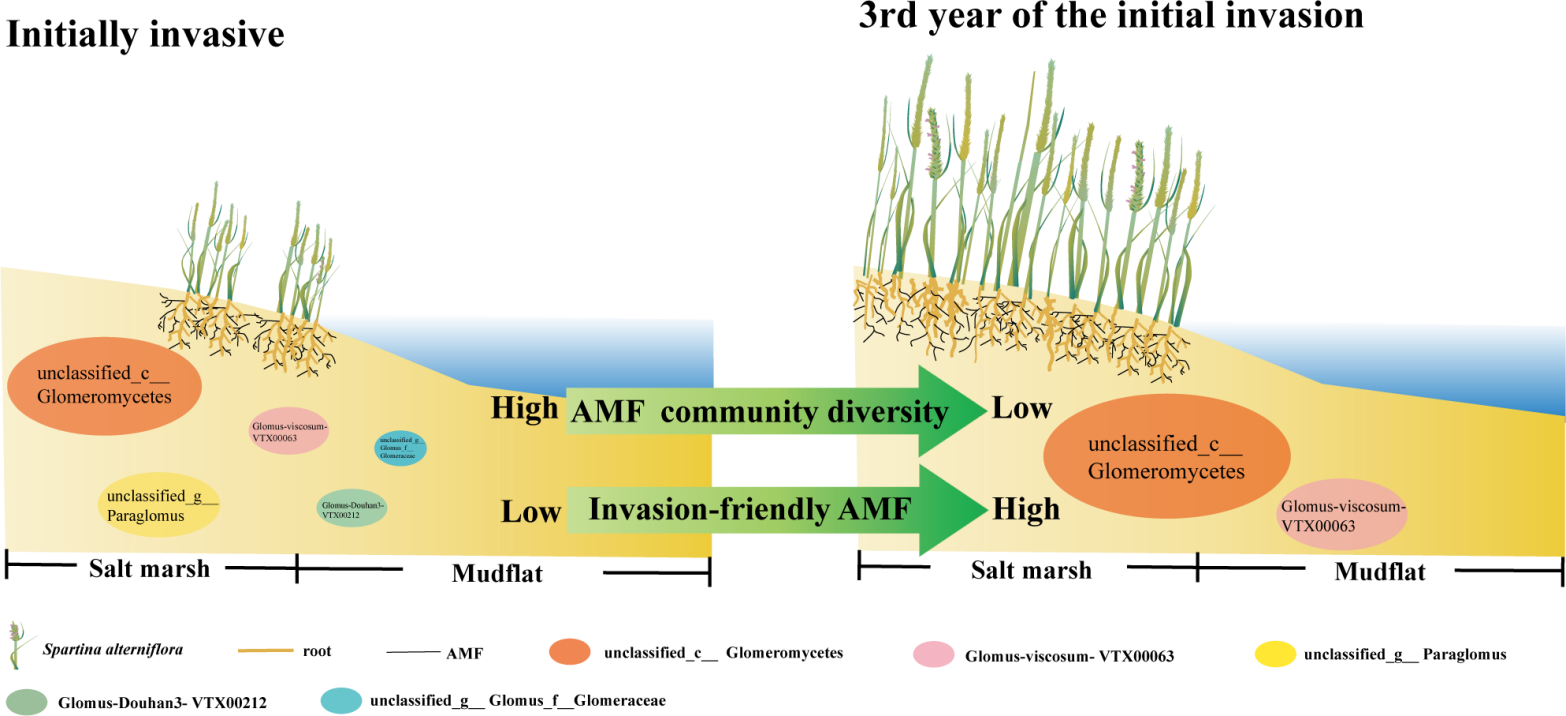
Potential mechanism of early *S. alterniflora* invasion reshaping soil AMF communities.

The introduction of exotic plants poses potential risks and challenges. In our study, soil physicochemical variables and soil AMF communities were investigated for initial introduction chronosequence of *S. alterniflora* in Chinese coastal salt marshes. The present results highlight the rapid and seasonal variation in short-term venation sequences of soil microbial communities to *S. alterniflora* introduction and support our hypothesis. Due to alternating seasons, AMF communities associated with *S. alterniflora* may be modified to some extent each year. During the 3-year introduction history, changes in soil AMF communities were closely related to changes in soil ω, AN, AK and N/P ratio, resulting in strong changes in microbial-mediated ecological functions. Research revealed that soil AMF underwent considerable dynamic succession along the time series process during the initial stage of *S. alterniflora* invasion. The introduction of *S. alterniflora* during the early stages of colonization triggers a homogenization of the AMF community, a phenomenon characterized by a notable reduction in soil AMF diversity without substantial alteration in evenness. The dominance of *unclassified_c__Glomeromycetes* and *Glomus viscosum-VTX00063* is observed to incrementally rise in the newly homogenized AMF assemblage. These shifts in AMF dominance patterns are indicative of the species’ adaptive response to *S. alterniflora* invasion, suggesting that the reshaped AMF community serves to bolster the competitive prowess of *S. alterniflora* amidst the surrounding vegetation (**Figure 7**). Soil available phosphorus (AP) positively affected important taxa of the early invading AMF community. Such alterations could be linked to *S. alterniflora*’s need for soil nutrients and its ability to adapt to the environment, potentially impacting the wetland ecosystem’s overall wellbeing and stability. Consequently, analyzing the dynamic succession of soil AMF, especially when introducing exotic plants, is vital for formulating efficient strategies for wetland conservation and rejuvenation.

## Data availability statement

The data presented in the study are deposited in the GenBank repository, accession number SAMN36708110-SAMN36708178, BioProject PRJNA998605.

## Author contributions

YJ: Writing – review & editing, Writing – original draft, Visualization, Validation, Software, Methodology, Investigation, Formal analysis, Data curation, Conceptualization. MW: Writing – original draft, Software, Investigation. XY: Writing – original draft, Supervision, Investigation. ML: Writing – original draft, Methodology, Investigation. XG: Writing – original draft, Writing – review & editing, Validation, Supervision, Resources, Project administration, Methodology, Funding acquisition, Data curation.
