# Supplementary material for: Exotic plants introduction changed soil nutrient cycle and symbiotic relationship with arbuscular mycorrhizal fungi in wetland ecological projects
Source: Front Plant Sci. 2024 Jul 10;15:1410009. doi: 10.3389/fpls.2024.1410009 (PMC11266298; doi:10.3389/fpls.2024.1410009)

Exotic plants introduction changed soil nutrient cycle and symbiotic relationship with arbuscular mycorrhizal fungi in wetland ecological projects

Yuxin Jiang[1], Mengxuan Wang[1], Xue Yan[1], Miaodan Liu[1], Xiaohong Guo[1*]

1 School of Resources and Environmental Engineering, Ludong University, Yantai, China

* Correspondence:

Xiaohong Guo

lddlgxh@126.com

Key word: Exotic plants introduction; Coastal wetland; *Spartina alterniflora*; Arbuscular Mycorrhizal Fungi; Plant invasion

Figure S1 Venn diagram depicting unique and shared OTUs between different interannual soils.

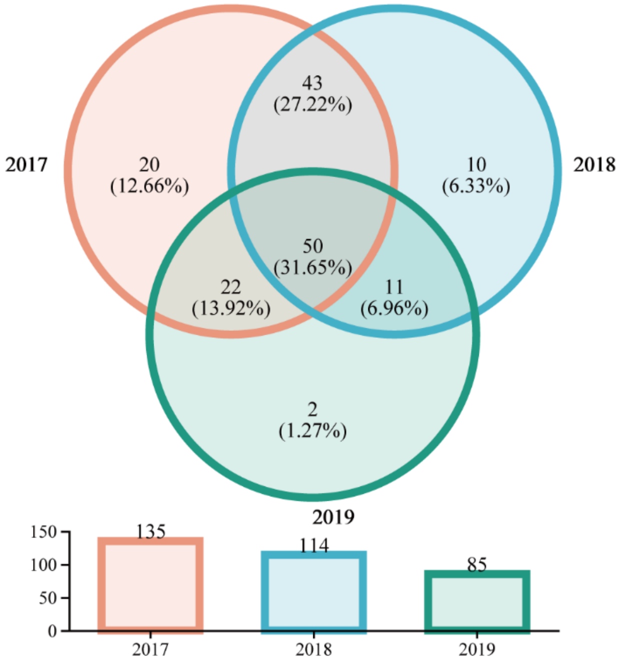

Figure S2 NMDS plots and fungal community differences of inter-annual sequence (A) and seasonality (B) at the genus level based on Bray-Curtis distance.

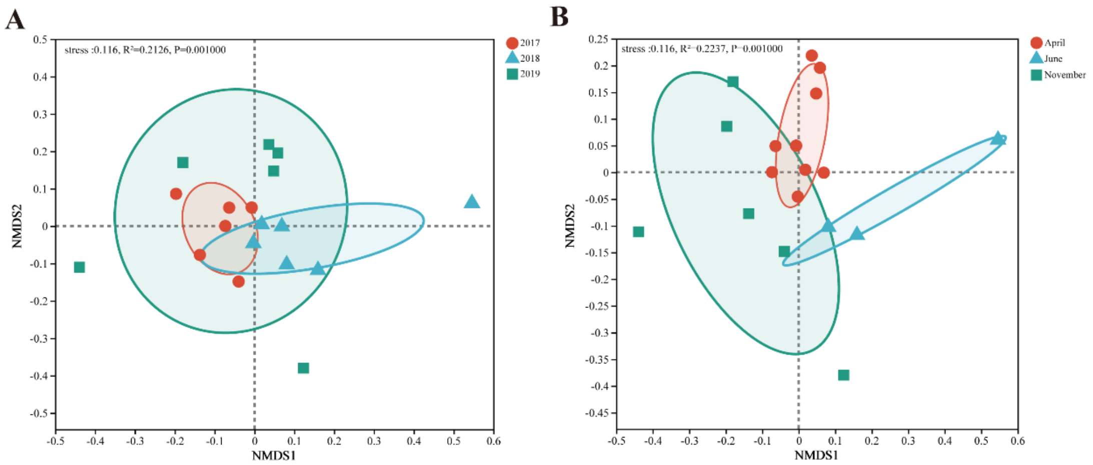

Supplement: Supplementary file 1 [file Image_1.pdf]
